# Catalpol enhanced physical exercise-mediated brain functional improvement in post-traumatic stress disorder model via promoting adult hippocampal neurogenesis

**DOI:** 10.18632/aging.203313

**Published:** 2021-07-29

**Authors:** Lina Sun, Weiwei Zhang, Ruiqi Ye, Lei Liu, Lili Jiang, Chao Xi

**Affiliations:** 1 School of Physical Education, Beijing Normal University, Beijing, China; 2 Department of Anesthesiology, Shanxi Bethune Hospital, Beijing, China; 3 School of Life Science, Beijing Normal University, Beijing, China

**Keywords:** catalpol, exercise, PTSD, hippocampal neurogenesis

## Abstract

Post-traumatic stress disorder (PTSD) is a serious psychiatric disorder characterized by hyper-response to environmental cues as well as the associated depressive and cognitive dysfunctions. According to the key roles of hippocampus for cognitive and emotional regulation, improving hippocampal functions, particularly hippocampal neural plasticity, is the necessary pathway to attenuate the core symptoms of PTSD. The effects of the alternative therapies such as exercise and natural compounds to reduce PTSD symptoms and promote adult hippocampal neurogenesis have been widely demonstrated. However, what is the effect of combining the exercise with traditional Chinese medical compounds remains unknown. In current study, we evaluated the effects of catalpol, which showed the pro-neurogenic effects in previous report, in regulating exercise-mediated PTSD therapeutic effects. With behavioral tests, we found that catalpol treatment promoted the effects of exercise to reduce the response of mice to dangerous cues, and simultaneously enhanced the antidepressant and cognitive protection effects. Moreover, by immunofluorescence we identified that catalpol promoted exercise-mediated hippocampal neurogenesis by enhancing the neural differentiation and mature neuronal survive. We further found that the promote effects of catalpol to exercise-induced environmental hyper-response, antidepressant effects and cognitive protective effects were all compromised by blocking neurogenesis with temozolomide (TMZ). This result indicates that hippocampal neurogenesis is prerequisite for catalpol to promote exercise-mediated brain functional improvement in PTSD model. In conclusion, our research identified the new function of natural compounds catalpol to promote the exercise-mediated brain functional changes in PTSD model, which depend on its effect promoting adult hippocampal neurogenesis.

## INTRODUCTION

Post-traumatic stress disorder (PTSD) is an anxiety disease that develops following frightening, stressful, or distressing life events, which affects the normal life and daily work of the subject with intense fear, helplessness, and stress. The behavioral dysfunction of the PTSD mainly resulted from the dysregulation of the hippocampus, which plays the primary role in regulating learning and memory as well as the emotional functions [1, 2]. Thus, one of the key steps to attenuate PTSD associated symptoms is the recover the functions of the hippocampus, particularly the hippocampal neural plasticity.

Adult hippocampal neurogenesis provides the structural plasticity to hippocampal networks to enhance the adaptability of animal to environmental stress [3]. In the dentate gyrus (DG) region of the hippocampus, neural stem cells (NSCs) experience the systematic biological events from proliferation, neural fate commitment, neural differentiation and maturation that form the newborn neurons to integrate into neural circuits [4]. The new formed neurons in DG region engage the hippocampus to separate the environmental cues and support the discrimination among the danger and safety surroundings [5]. During the PTSD development, the over-stress from the trauma resulted in the hyper-response of the subjects and caused the systematic stress reaction such as the hyperactive hypothalamic-pituitary-adrenal (HPA) axis and immune response [6, 7]. Most of the stress response could affect the decreased adult hippocampal neurogenesis and thereby further decreased the pattern separation to environmental cues. The vicious cycle induces the anxiety and depressive behavior to normal live stimulations. Moreover, the decreased adult hippocampal neurogenesis also suppress the learning and memory of the animals [8]. Thus, improving the adult neurogenesis is the key to explore the therapeutic methods to PTSD.

Physical exercise has been widely reported as the effective and less-cost remedy to PTSD [9]. Moreover, exercise could improve the hippocampal neural plasticity and reduce the depressive/anxiety mood and enhance the cognitive functions [10, 11]. In our previous work, we have identified the effects of exercise in reduce PTSD associated depression and anxiety Akt-mediate regulating adult hippocampal neurogenesis [12]. But how to optimize the therapeutic effects of exercise still requires further exploration.

Traditional Chinese Medical herbs contains enormous types of compounds with effective neuroprotection. Catalpol is widely reported as its antidepressant effects as well as the pro-neurogenic functions [13, 14]. It was demonstrated that catalpol could perform the antidepressant functions via the BDNF dependent mechanism, which also contributes the neurogenesis regulation in hippocampus [14]. More importantly, catalpol could improve the learning and memory behaviors in cognitive disorder models including attention deficit hyperactivity disorder (ADHD), Parkinson’s disease and Alzheimer’s disease [15, 16]. However, whether catalpol could perform thet therapeutic effects in PTSD in combined with physical exercise remains unknown. Furthermore, what is the role of adult hippocampal neurogenesis in the effects of catalpol to brain functional improvement still not fully understanding.

In this study, in order to test the effects of catalpol to exercise-mediated PTSD therapy, we created the disease animal model and discovered that catalpol could perform as the synergetic effects to attenuate fear condition response, improve antidepressant and cognitive behaviors. Moreover, we also found that adult hippocampal neurogenesis is required by the effects of catalpol to promote the exercise-mediated therapeutic effects to PTSD.

## MATERIALS AND METHODS

### Animals

8 weeks adult C57BL/6N male mice were obtained from Laboratory Animal Centre in Beijing Normal University. Mice were raised with ad libitum of tap water as well as food pellets during whole experimental period. All animal was housed in a 12:12 h light/dark cycle. Animal experiment was approved by ethical committee in Beijing Normal University in regard of Guidelines for the Care and Use of Mammals in Neuroscience and Behavioral Research (2003). Temperature (21.6°C) and humidity (24%) were monitored and maintained at a constant level throughout the experiment. To induce the PTSD symptoms, unconditional stimulation combined with conditional stimulation (US-CS) was first performed by pulse the mice with electronic stimulation with a electric foot shock for 5 min (1.5mA) coupled with the conditional noise. In the rest of the day, mice are exposed to noise as CS and the freezing response was recorded. No foot shock was performed for control group during US. Treadmill exercise was performed with the protocol of 20 m/min treadmill (30 min/day). Catalpol treatment was performed with the IP injection with dosage of 20 mg/kg.

For labelling the proliferating cells in hippocampus, BrdU was injected to the mice with the dosage of 100 mg/kg once before the sacrifice 24 h. For labelling the newborn mature neurons in hippocampus, BrdU was daily injected with dosage of 50 mg/kg for 3 days 20 days previous sacrifice.

To suppress adult neurogenesis, Temozolomide (TMZ, Sigma Aldrich) was given to mice at 25 mg/kg/d (IP, 2.5 mg/ml in saline), and the injections were performed for 4 days previous of the US-CS induction.

### Behavioral tests

PTSD symptoms was evaluated by recording the freezing time of the mice after the CS. The antidepressant/anxiety behavior, and cognitive behavior were analyzed by following procedure:

Depressive symptoms were assessed by forced swim test (FST) and tail suspension test (TST). Briefly, mice were put into a cylinder water tank (30 cm height × 20 cm diameters) with the water temperature 23–25°C for 6 min. The mobility time within last 4 min (define the first 2 min as habituation) including struggling and free swim time were recorded for analyzing. For TST, mice were suspended on the tap-top of their tails for 6 min. Distance from mice nose to the floor was 25 cm. The mobility time of mice were recorded for analyzing their learned helplessness. Open field test (OFT) and elevated plus maze (EPM) were employed for testing the anxiety mood. Mice were put into an open field with the size of 43 cm × 43 cm for 10 min. Duration of the animal traveled in the central area was recorded to analyze the anxiety level. Open field would be cleaned with ethanol before the test. For EPM, the apparatus used was a platform comprises two open arms (25 × 5 × 0.5 cm) across from each other and perpendicular to two closed arms (25 × 5 × 16 cm) with a center platform (5 × 5 × 0.5 cm). Mice were processed to move freely about the maze for 10 min. The time of the mice staying on open arm were recorded for analyzing the anxiety- behavior.

Morris water maze (MWM) and objective recognition tests were following our previous publication. In MWM, navigation test was performed by settle the platform under the surface of the water in target zone. Mice were processed to free swim and position the platform with 120 sec. After 3 training days, platform was moved to the opposite zone to detect the memory retrieve of the mice. Escape latency was daily recorded for evaluating spatial learning. In last day of the test, the hidden platform was removed, and mice were processed to free swim in the pool for 120 sec. Swim time in the target zone, where the platform was, was recorded to evaluate the spatial memory.

### Immunofluorescence

Mice were conducted with cardiac perfusion by 4% paraformaldehyde (PFA). Brain was collected and post-fixed in 4% PFA for 24 h. Tissue was then embedded after dehydration with 30% of sucrose. Hippocampal cryosections were prepared. Briefly, sections after antigen retrieve (pH 6.0 citrate acid) and washing with 0.3% TritionX-100 PBS were incubated with 5% goat serum (0.3 TritionX-100). For BrdU staining, sections were incubated with 2M HCl for 1 h at room temperature (RT) and wash with PBST for 3 times (15 min each time). Primary antibodies (Rabbit-anti-NeuN, CST, 1:200; Mouse-anti-BrdU, CST, 1:400; Rabbit-anti-DCX, CST, 1:400; Rabbit-anti-MCM2, CST, 1:400) were incubated with sections at 4°C overnight. Secondary antibodies (Goat-anti-Rabbit-Alexa fluor 488, ThermoFisher, 1:800; Goat-anti-Mouse-Alexa fluor 568, ThermoFisher, 1:800) were incubated with sections for 2 h RT. DAPI (Sigma) was incubated with sections for 10 min. Tissue IF images were obtained with confocal microscopy (Carl Zeiss, LSM 700) with Z-stack for 20 μm. Image was shown as maximum projection of Z-stack. Required positive cell number was calculated and displayed as the cell density per mm^2^.

### Data availability statement

Data are available with reasonable requirements.

### Statistical analysis

All data were showed with Mean SEM. Statistical analysis was conducted with graph pad Prism 8. One-way ANOVA was used for comparing the multiple groups with one factors. Two-way ANOVA was used for comparing groups interacted with two factors. Tukey post-how analysis was performed after ANOVA comparing. Student *t*-test was used for comparing two groups. A *p* value of 0.05 was cut as significance.

## RESULTS

### Combination of catalpol enhanced the effects of treadmill to reduce PTSD symptoms

**Figure 1:**
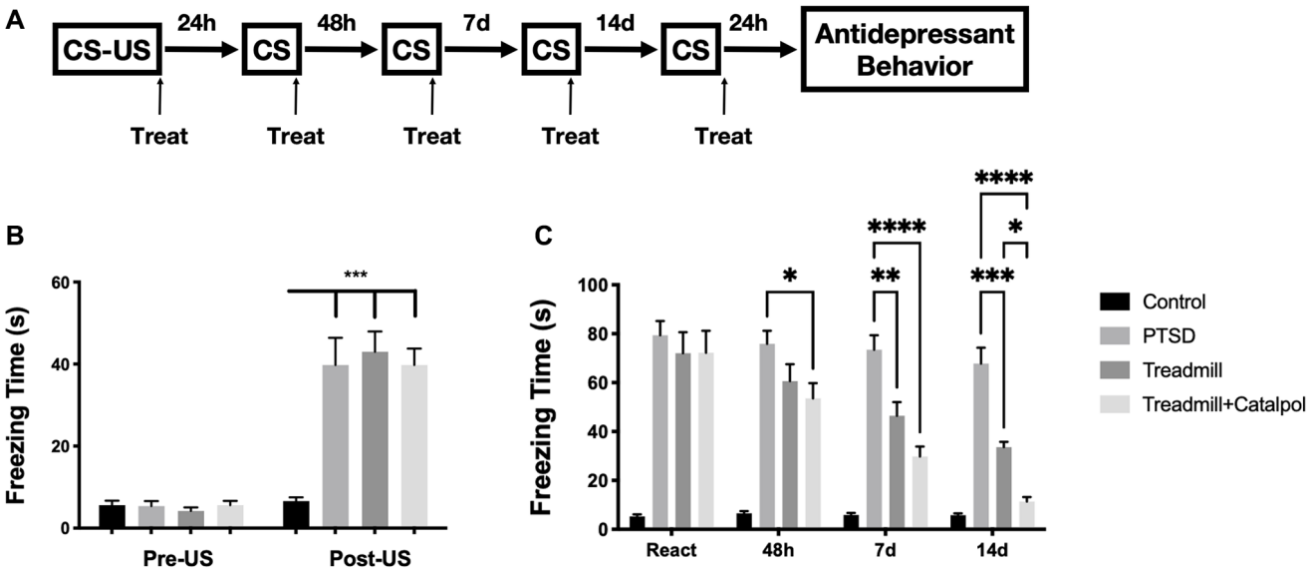
**Combination of catalpol enhanced the effects of treadmill to reduce PTSD symptoms.** (**A**) Experimental process to establish the CS-US induced PTSD behavioral dysfunctions. (**B**) Freezing time comparation before and after the US performance. (**C**) Daily recorded freezing time under CS stimulation in different treatment. Two-way ANOVA, Tukey’s post-hoc, ^*^p < 0.05, ^**^p < 0.01, ^***^p < 0.001, ^****^p < 0.0001.

We used the fear learning to mimic the development of the PTSD. The conditioned stimulus combined with unconditioned stimulus (US) were firstly performed for establishing the panic memory of the animal. Then the repeat conditioned stimulus (CS) to mimic the post-traumatic stress disorder [17]. The treadmill and treadmill combined with catalpol administration were performed right after each CS. After 4 times of the CS and the treatment, the mice were then performed with the antidepressant and cognitive tests (Figure 1A). Compared with CS, the freezing time of the mice after US showed the dramatic increase relative with control group (Figure 1B, two-way ANOVA, Tukey’s post-hoc test, PTSD vs. Control: *p* < 0.001). Such result indicates the successful establishment of the PTSD model. Following the CS sections after US, the catalpol was administrated to the mice with oral gavage previous treadmill. Treadmill combined with catalpol administration displayed the significant therapeutic effects to decrease the freezing time after 48 h, and such effect continued until the end of the CS protocol (Figure 1C, two-way ANOVA, Tukey’s post-hoc test, treadmill + catalpol vs. PTSD: *p* < 0.05 at 48 h, *p* < 0.001 on day 7 and day 14). Although treadmill administration alone also present the attenuating effects to freezing time, the significance did not show 48 h after the US. Moreover, on day 14 the freezing time also remarkably longer than combination group (Figure 1C, two-way ANOVA, Tukey’s post-hoc test, treadmill vs. PTSD: *p* < 0.01 on day 7 and day 14; treadmill vs. treadmill + catalpol: *p* < 0.05 on day 14). Collectively, catalpol treatment significantly promote the effects of the treadmill running in attenuating PTSD syndrome.

### Combination of catalpol promoted the effects of treadmill to reduce PTSD associated emotional and cognitive dysfunctions

**Figure 2:**
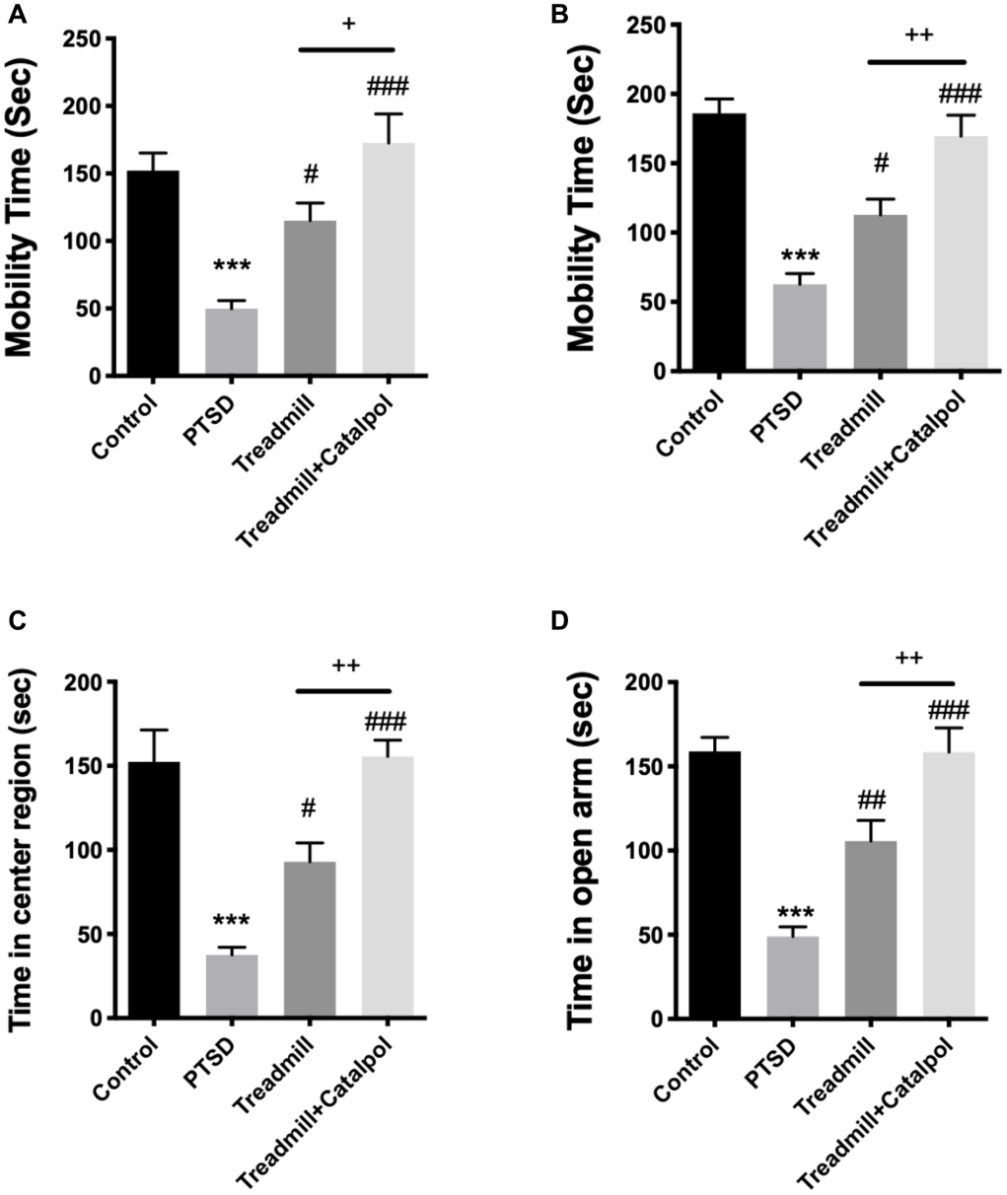
**Combination of catalpol promoted the effects of treadmill to reduce PTSD associated depressive and anxiety behaviors.** (**A**, **B**) Mobility time of different groups in FST and TST. (**C**) Time of the mice for staying in the center region in OFT. (**D**) Time of the mice for staying in the open arm during EPM. One-way ANOVA, ^***^p < 0.001 vs. control; ^#^*p* < 0.05, ^###^*p* < 0.001 vs. PTSD; ^+^*p* < 0.05, ^++^*p* < 0.01 vs. exercise treatment group.

Since PTSD also results in serious dysfunctional antidepressant ability of the subjects, we further tested the depression/anxiety behaviors after the CS tests. As the result showed, the mobility time of the mice were significantly prolonged in treadmill as well as the combined treatment group in both FST and TST (Figure 2A, 2B, one-way ANOVA, FST: treadmill vs. PTSD, *p* < 0.05; treadmill + catalpol vs. PTSD: *p* < 0.001; TST: treadmill vs. PTSD, *p* < 0.05; treadmill + catalpol vs. PTSD: *p* < 0.001). Compared with the treadmill administration alone, mice treated with treadmill training in combined with the catalpol also showed the remarkable improvement of the mobility (Figure 2A, 2B, one-way ANOVA, FST: treadmill vs. treadmill + catalpol: *p* < 0.05; T ST: treadmill vs. treadmill + catalpol: *p* < 0.01). Herein, catalpol could enhance the antidepressant effects of treadmill in PTSD model. On the other side, OFT and EPM were performed to test the anxiety behaviors of the mice. In consistent with the result in FST and TST, the mobility time of the mice in treadmill training plus catalpol treatment and treadmill treatment alone resulted in the significant increasing in compared with PTSD (Figure 2C, 2D, one-way ANOVA, OFT: PTSD vs. treadmill: *p* < 0.05; PTSD vs. treadmill + catalpol: *p* < 0.001; EPM: PTSD vs. treadmill: *p* < 0.01; T PTSD vs. treadmill + catalpol: *p* < 0.001). Notably, catalpol also significantly improved the anxiolytic effects of treadmill that reflected by the obvious increased time in center region of OFT and the open arm in EPM (Figure 2C, 2D, one-way ANOVA, OFT: treadmill + catalpol vs. treadmill: *p* < 0.01; EPM: treadmill + catalpol vs. treadmill: *p* < 0.01). Collectively, catalpol exerts the promotive effects to exercise-induced antidepressant and anxiolytic functions.

**Figure 3:**
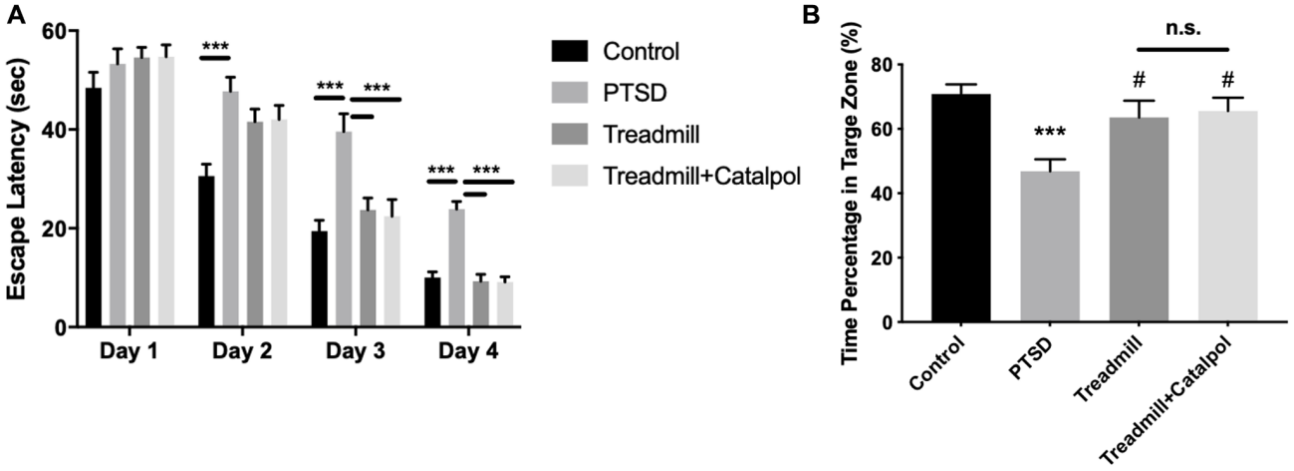
**Combination of catalpol promoted the exercise-induced cognitive protection in PTSD mice.** (**A**) The latency escape time of the mice in acquisition test. (**B**) Time percentage of the mice to swim in the target zone during the probe trail. Two-way ANOVA for acquisition test, ^*^p < 0.05, ^**^p < 0.01, ^***^p < 0.001. One-way ANOVA for probe trail, ^***^p < 0.001 vs. control; ^#^*p* < 0.05 vs. PTSD.

Given the cognitive dysfunction is also considered as the combined symptom associated with PTSD [18], we further tested the learning and memory ability with Morris water maze. In acquisition task, which aimed to test the spatial learning of the mice, PTSD mice displayed the dramatic prolonged escaping time to position the hidden platform (Figure 3A, two-way ANOVA, Tukey’s post-hoc test, PSTD vs. control: *p* < 0.001 on Day 2, Day 3 and Day 4). Along with the hidden platform training, mice conducted the treadmill remedy showed the significant shorter acquisition time from Day 3 (Figure 3A, two-way ANOVA, Tukey’s post-hoc test, PSTD vs. treadmill: *p* < 0.001 on Day 3 and Day 4). However, mice conducted with the treadmill and catalpol treatment showed the significance right Day 2 (Figure 3A, two-way ANOVA, Tukey’s post-hoc test, treadmill + catalpol vs. treadmill: *p* < 0.001 on Day 2, Day 3 and Day 4). Compared with treadmill administration alone, catalpol treatment also resulted in the remarkable shorter acquisition time during the acquisition training (Figure 3A, two-way ANOVA, Tukey’s post-hoc test, treadmill + catalpol vs. treadmill: *p* < 0.01 on Day 3 and Day 4). After acquisition task, we further tested the spatial memory of the mice by removing the platform. In probe trail, mice treated with treadmill alone and treadmill plus catalpol had the significant swim time compared with PTSD (Figure 3B, one-way ANOVA, PTSD vs. treadmill: *p* < 0.05; PTSD vs. treadmill + catalpol: *p* < 0.05). However, the spatial memory between these two groups showed no significant difference. The possible reason could be the already established memory in acquisition task. Even though, it could also be concluded that catalpol could promote the effects of the treadmill training to improve cognitive functions.

### Catalpol promoted the neurogenic improvement effects of the treadmill by accelerating neural differentiation

**Figure 4:**
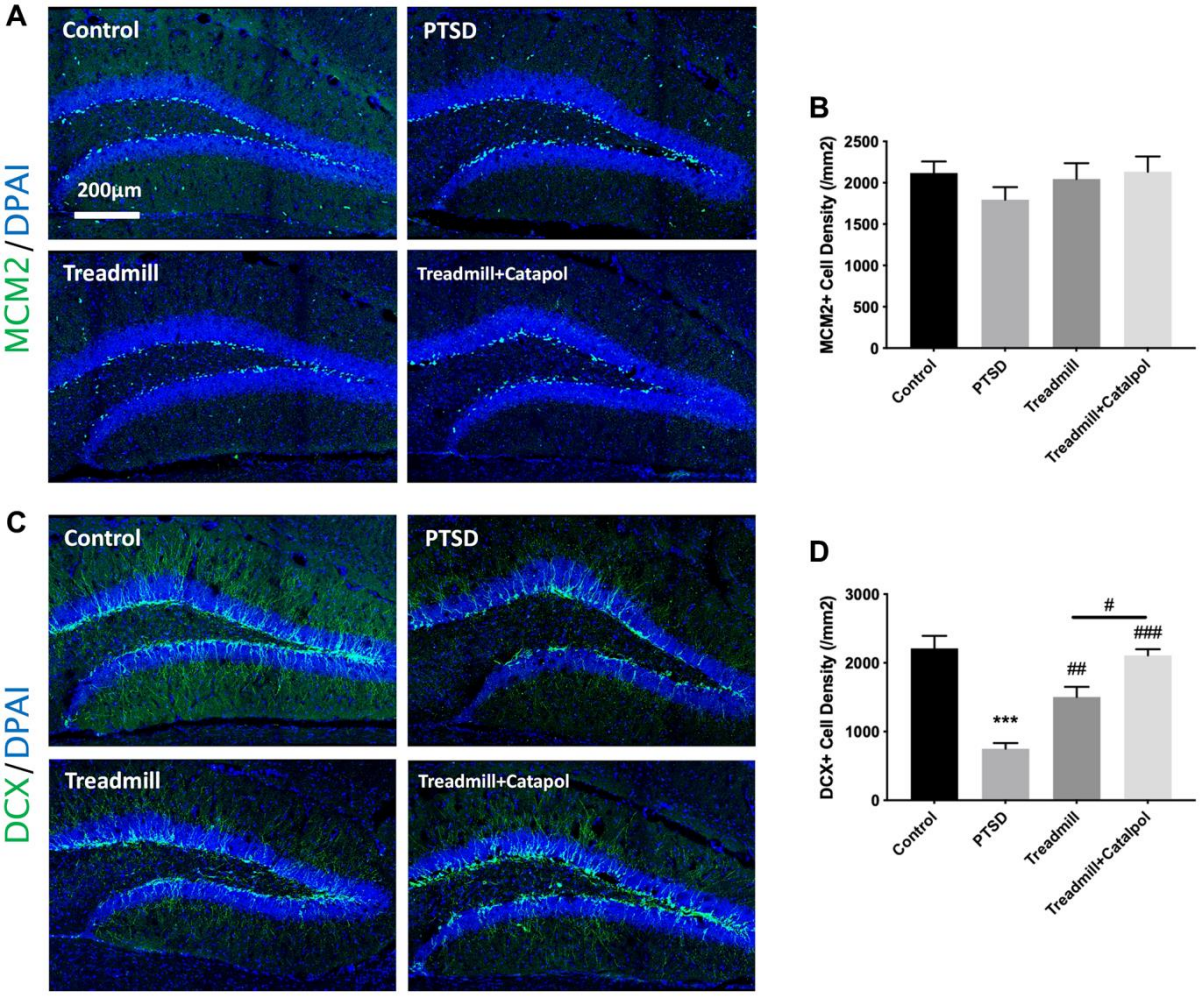
**Catalpol promoted the neurogenic improvement effects of the treadmill by accelerating neural differentiation.** (**A**) MCM2 staining (green) in DG to assess the NSCs proliferation. DAPI was used for unclear labelling. (**B**) No significant difference showed among groups for MCM2 positive cell density. (**C**) DCX staining (green) in DG to assess the NSCs neural differentiation. DAPI was used for unclear labelling. (**D**) Statistical analysis of the DCX positive cell density in different groups. One-way ANOVA, ^***^p < 0.001 vs. control; ^#^*p* < 0.05, ^##^*p* < 0.01, ^###^*p* < 0.001.

Hippocampal neurogenesis is the key physiological mechanism underlying the emotional and cognitive behaviors [19]. More importantly, the effects of exercise in pro-neurogenesis have been widely reported [20]. Therefore, we further tested the proliferation of NSCs and the neural differentiation by immunofluorescence. We first tested the proliferation profile in the hippocampal DG region. The active NSCs and NPCs, which are almost counted as the proliferating cell types in hippocampus, could be considered as the potential value to reflect the hippocampal neurogenesis. By staining the proliferating cells with MCM2, we detected that the proliferating cells among all treatments showed no significance (Figure 4A, 4B; one-way ANOVA). Doublecortin (DCX) is the biological marker of the immature neurons and neural progenitor cells. We then labelled the hippocampus with DCX for testing the profile of the neural differentiation. As the result indicated, PTSD caused the dramatic decreased DCX density in comparing with control (Figure 4C, 4D; one-way ANOVA, PTSD vs. control: *p* < 0.001). In compared with PTSD model, the treadmill administration improved the density of the DCX positive immature neurons in hippocampus (Figure 4C, 4D; one-way ANOVA, PTSD vs. treadmill: *p* < 0.01). While the catalpol combined with treadmill administration resulted in the significant enhanced increase of the DCX positive neural density (Figure 4C, 4D; one-way ANOVA, treadmill + catalpol vs. treadmill: *p* < 0.001). Based on the effects of catalpol and exercise combination did not improve the NSCs/NPCs proliferation in significant level, the increased DCX positive neural resulted by catalpol as well as the treadmill could be suggested by their effects in promoting the neural fate choice and differentiation of the NSCs.

### Catalpol promoted neural differentiation without changing the survive of the immature neurons

**Figure 5:**
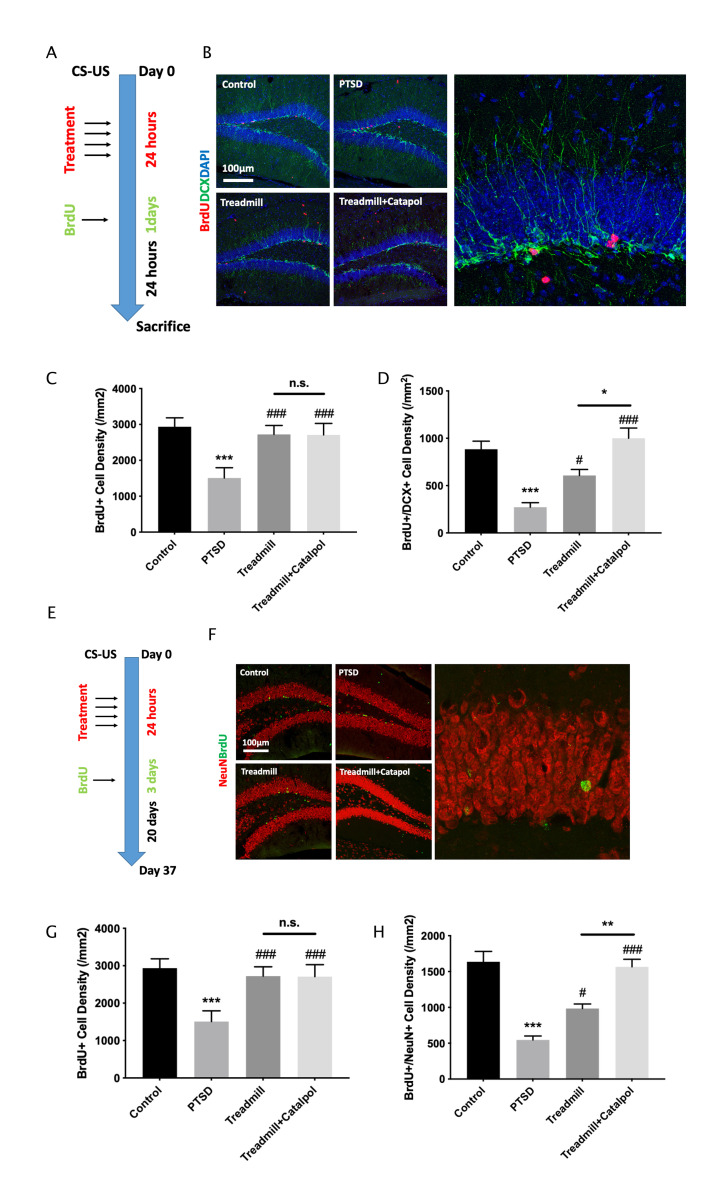
**Catalpol promoted neural differentiation without changing the survival of the immature neurons.** (**A**, **E**) experimental procedure of different BrdU injection protocol. (**B**) DCX staining (green) coupled with BrdU (red) in DG to assess the NSCs neural differentiation. (**C**, **D**) Statistical analysis of the BrdU positive cell and BrdU/DCX dual positive cells in DG region. (**F**) NeuN staining (red) coupled with BrdU (green) in DG to assess the neural maturation in DG. (**G**, **H**) Statistical analysis of the BrdU positive cell and BrdU/NeuN dual positive cells in DG region. One-way ANOVA, ^***^p < 0.001 vs. control; ^#^*p* < 0.05, ^###^*p* < 0.001.

The new formed neurons in hippocamapus could be resulted in the increased neural differentiation as well as the enhanced survive of the new formed mature neurons. To confirm which effects is the main biological mechanism of the catalpol induced exercise therapy promotion, we further labelled the hippocampal new formed cells by injecting BrdU with different interval durations. For testing the immature neuron formation at hippocampal region, mice were treated the exercise and catalpol were performed 4 time in 1 day after the CS-US and injected the BrdU once (100 mg/ml) 24 h previous the sacrifice (Figure 5A). Compared with PTSD model, treadmill administration improved both the density of BrdU^+^ cell as well as the BrdU and DCX dual positive cell (Figure 5B–5D; one-way ANOVA, BrdU^+^, treadmill vs. PTSD: *p* < 0.05; BrdU^+^/DCX^+^, treadmill vs. PTSD: *p* < 0.05). Compared with the treadmill administration alone, catalpol combined with exercise treatment increased the BrdU^+^ cell density as well as the BrdU and DCX dual positive cell density (Figure 5B–5D; one-way ANOVA, BrdU^+^, treadmill vs. catalpol + treadmill: *p* < 0.05; BrdU^+^/DCX^+^, treadmill vs. catalpol + treadmill: *p* < 0.05). For further test the survive of the new formed mature neurons, mice were treated the exercise and catalpol were performed 4 time in 1 day after the CS-US and injected the BrdU 3 days (100 mg/ml/d) 10 days previous the sacrifice (Figure 5E). The BrdU and mature neuron marker NeuN were stained for detecting the new formed mature neurons. As the result indicated, although treadmill and treadmill combined with catalpol both improved the density of the BrdU^+^ cell as well as the BrdU and NeuN dual positive cell (Figure 5F–5H; one-way ANOVA, BrdU^+^, treadmill vs. PTSD: *p* < 0.001; BrdU^+^/NeuN^+^, treadmill vs. PTSD: *p* < 0.001), catalpol treatment did not result in any significant difference on the density of BrdU^+^ cell in compared with treadmill treatment (Figure 5F–5H; one-way ANOVA). While treatment of catalpol combined with the treadmill administration dramatically increased the density of BrdU^+^/NeuN^+^ cells (Figure 5F–5H; one-way ANOVA, BrdU^+^/NeuN^+^, treadmill vs. treadmill + catalpol: *p* < 0.01). Collectively, the treatment of the catalpol could enhance both neural differentiation as well as the survive of the new mature neurons in hippocampus.

### Adult neurogenesis is required for catalpol to promote exercise therapeutic effects to panic memory retrieve and antidepressant

**Figure 6:**
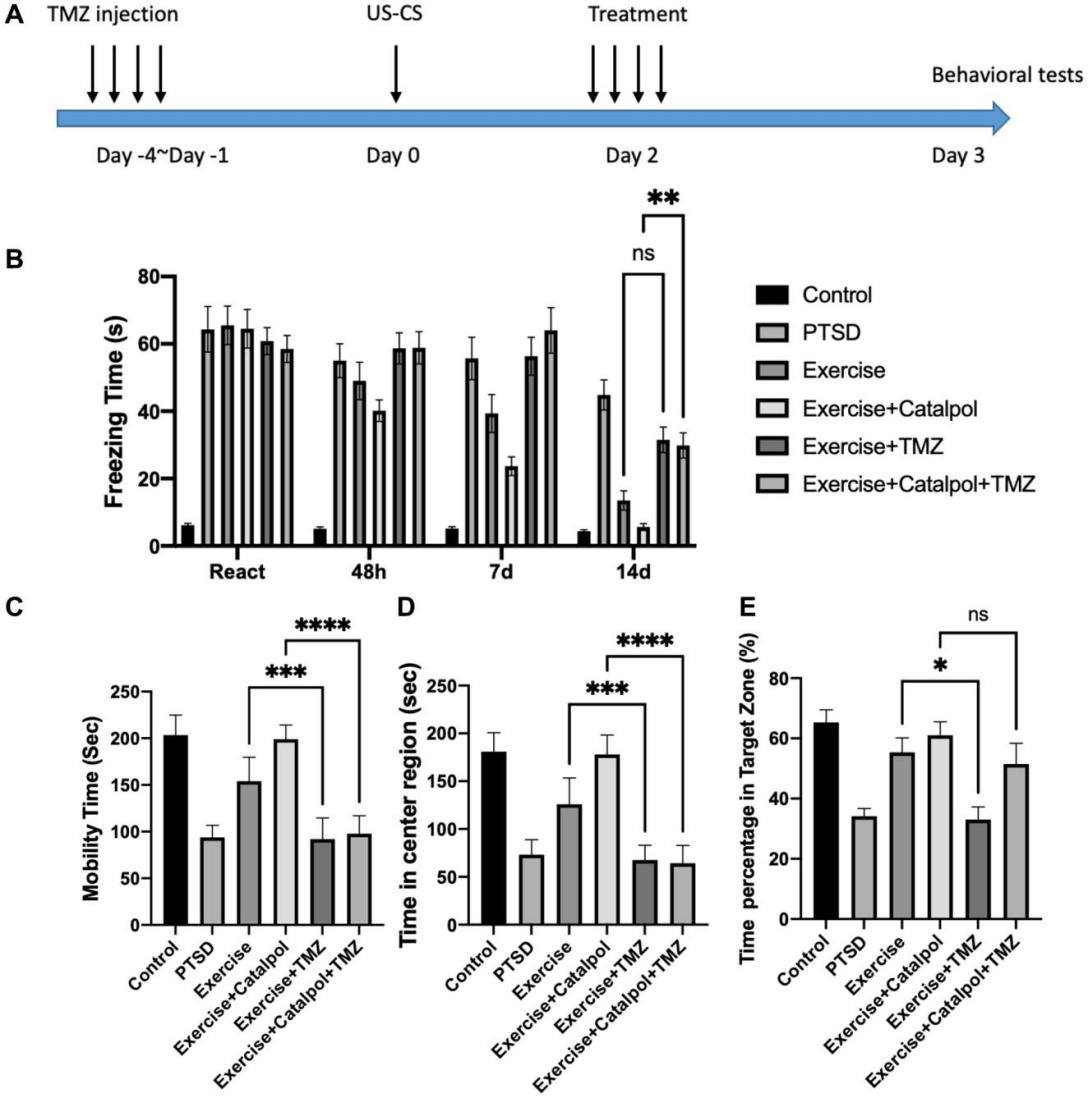
**Adult neurogenesis is required for catalpol to promote exercise therapeutic effects to panic memory retrieve and antidepressant.** (**A**) Experimental procedure to block the adult hippocampal neurogenesis with TMZ injection. (**B**) Daily freezing time response change among different treatments. (**C**) Statistical analysis of the mobility time in FST among different treatments. (**D**) Statistical analysis of the staying time at center region in OFT among different treatments. (**E**) Statistical analysis of the swimming time at target zone in probe trail among different treatments. One-way ANOVA, ^*^p < 0.05, ^***^p < 0.001, ^****^p < 0.0001.

To further analyze the role of adult neurogenesis in regulating effects of catalpol to exercise mediate PTSD therapy, we injected the mice with TMZ to block the cell cycle of the NSCs (Figure 6A). After the treatment of TMZ, we observed that the attenuating effects of exercise combined with catalpol to decrease the CS was compromised (Figure 6B, two-way ANOVA, Tukey’s post-hoc test, 14d catalpol + exercise vs. catalpol + exercise + TMZ: *p* < 0.001). While no significance showed after TMZ treated with the exercise group. In contrast, the effects of exercise to improve the mobility in FST and to prolong the activity in center region in OFT were all prohibited by treatment of the TMZ (Figure 6C, 5D, one-way ANOVA, Tukey’s post-hoc test, FST, exercise vs. exercise + TMZ: *p* < 0.001; OFT, exercise vs. exercise + TMZ: *p* < 0.001). Moreover, TMZ also suppressed the effects of catalpol combination effects in regulating exersise-mediated antidepressant and anxiolytic functions (Figure 6C, 6D, one-way ANOVA, Tukey’s post-hoc test, FST, exercise + catalpol vs. exercise + catalpol + TMZ: *p* < 0.001; OFT, exercise + catalpol vs. exercise + catalpol + TMZ: *p* < 0.001). Additionally, we employed the MWM to test the cognitive behavior. After the hidden platform acquisition, in probe trail TMZ treatment dramatically decreased the effects of the exercise in prolong the time of the swim in target zone (Figure 6E, one-way ANOVA, Tukey’s post-hoc test, exercise vs. exercise + TMZ: *p* < 0.001). However, such effects did not detect in the TMZ treated catalpol combined with exercise (Figure 6E). This provides the evidence that neurogenesis might only plays the critical roles in regulating the effects of exercise to improve panic memory response and emotional functions. Nevertheless, the working memory that regulated by catalpol combined with exercise was not totally depend on adult hippocampal neurogenesis.

## DISCUSSION

PSTD is the serious psychiatric issue to affect the daily life of the patients and bring the serious burden to the families. The treatment of the PTSD should not only focus on release the panic memory but also need to attenuate the associated emotional and cognitive symptoms. Exercise as well as the natural compounds from traditional herbs are two main alternative remedy strategies of antidepressants. However, whether combination of these two methods could results in the synergetic effects to PTSD treatment remains seldom explored. In this study, we detected the function of the catalpol to enhance the effects of the exercise-mediated behavioral improvement. Moreover, our study detected the pro-neurogenesis of the catalpol is the key mechanism underlying its synergetic functions to exercise-mediated PTSD therapy.

Fear-based disorders, like social anxiety disorder (SAD) and PTSD, are characterized by an exaggerated fear response and avoidance to trigger cues [21]. Thus, in this study we selected the US-CS coupling methods to create the PTSD pathology. The panic memory with the trigger cues was successfully established through US administration coupling with CS (Figure 1). Based on the core symptoms of the PTSD patients in sensitive avoidance behavior to the trigger cues, the response of the mice to the CS in following days could be utilized as the reliable value for therapeutic evaluation. In our study, we tested combination of the catalpol enhanced the effect of the exercise in attenuating the response behavior to CS (Figure 1). Such result provided the direct evidence to support the therapeutic effects of catalpol against the core symptom of the PTSD. Moreover, catalpol combination also promoted the antidepressant and cognitive improvement functions of exercise to PTSD model. Collectively, catalpol treatment could significant promote the behavioral effects of the exercise in attenuating PTSD symptoms.

Emotional and cognitive dysfunctions are two main aspects that associated with the PTSD. More importantly, the adult neurogenesis plays the critical role in regulating the emotional and cognitive behaviors, as well as the adaptability to environmental stress [22, 23]. From the behavioral tests, we confirmed that catalpol also promotes the antidepressant effects as well as the cognitive improvement of the exercise to PTSD mice model (Figures 2, 3). The result suggested the effects of catalpol in regulating exercise-induced learning and emotional functions improvements. Based that therapeutic effects of PTSD patients requires the systematic brain functions, our results suggests that catalpol could be applied as the synergetic supplements for treating PTSD combined with physical exercise.

Adult neurogenesis is the key neurobiological mechanism of the panic memory adaptation. Moreover, the enhancement of the exercise to improve adult hippocmapal neurogenesis have been widely reported as the fundamental of the exercise-mediated antidepressant function. It was reported that in stroke model combination of catalpol and puerarin effectively increase the angiogenesis in cortex and neurogenesis in hippocampus [24]. However, whether catalpol perform the neurogenic promoting effects in PTSD model remains unidentified. In this study, we detected that exercise-induced neurogenesis was dramatically enhanced by catalpol treatment. Increasing of the neurogenesis usually derived from the hyperproliferation of NSCs, enhanced neural differentiation as well as the increased survival rate of newborn neurons. In current study, we used MCM2 staining to confirm that catalpol and exercise exerts no significant effects to the proliferation of the NSCs (Figure 4). While the increased DCX staining induced by catalpol and exercise indicates that the treatment could enhance the neural differentiation of the NSCs. Therefore, we further labelled BrdU as the probe to assess whether enhancement of survive also contributes the effects of catalpol and exercise-mediated PTSD model (Figure 5). As the result indicated, prolonged BrdU labelling strategy obtained increased BrdU and NeuN dual positive labelling in PTSD mice treated with catalpol combined with exercise, and such treatment showed the improvement compared with exercise administration alone (Figure 5). These results indicate that catalpol also promote the survival of the newborn neurons in the hippocampal region.

TMZ induced neurogenic blockage is the well-established methods to assess the role of the adult neurogenesis to animal behavior [25, 26]. By suppressing the proliferation of the NSCs, TMZ treatment enables us to detect the role of the adult neurogenesis in effects of catalpol to PTSD model. In the results, we observed that TMZ treatment did not affect the fear condition response of the animal that administrated by exercise alone (Figure 6). However, TMZ caused the obvious prolonged freezing time when animal was treated with exercise combined with catalpol (Figure 6). Such results not only suggest that the effects of exercise to reduce PTSD symptom includes but not fully depends on adult neurogenesis, but also indicate that catalpol promote the exercise mediate PTSD therapy via promoting adult neurogenesis. In antidepressant behavioral tests, TMZ treatment blocked the effects of the exercise administration alone as well as the combination group (Figure 6). These tests indicate that the critical role of the adult neurogenesis in regulating antidepressant behaviors. In cognitive test, the memory of the mice that treated with exercise alone was blocked by TMZ, but such phenotype was not showed in the catalpol combined group (Figure 6). Therefore, the level of the dependence of catalpol to adult neurogenesis might fluctuate among different behavior patterns.

During the clinical treatment of the PTSD, alternative therapies could gain enormous benefits to improve the behavioral outcomes of the patients. However, the non-drug treatment particularly exercise have its limitation such as the big variation of the therapeutic effects as well as the unexpected stress to subjects [27]. Thus, combination with supplementary treatment such traditional Chinese medicine should be necessary. In this study, we aims to resolve if combine the exercise with herbal compound catalpol could have improvement to PTSD mice. For further study, we will continue to see how catalpol could attenuate the side effects from the exercise treatment. In conclusion, our current study indicates that catalpol could act as the synergetic agent to promote the behavioral improvement of exercise to PTSD subjects including the fear memory retrieve, depressive/anxiety symptoms and cognitive performance. Such behavioral functions mainly depend on the biological effects of the catalpol to enhance the adult hippocampal neurogenesis mainly in accelerating the neural differentiation of NSCs as well as in increasing the newborn neurons survive. This research also provides the insight to apply the natural compounds as the enhancer or supplement to combine with the alternative therapy to psychiatric disorders.
