# Correction for: Catalpol enhanced physical exercise-mediated brain functional improvement in post-traumatic stress disorder model via promoting adult hippocampal neurogenesis

**DOI:** 10.18632/aging.204873

**Published:** 2023-06-30

**Authors:** Lina Sun, Weiwei Zhang, Ruiqi Ye, Lei Liu, Lili Jiang, Chao Xi

**Affiliations:** 1 School of Physical Education, Beijing Normal University, Beijing, China; 2 Department of Anesthesiology, Shanxi Bethune Hospital, Taiyuan, China; 3 School of Life Science, Beijing Normal University, Beijing, China

**Keywords:** catalpol, exercise, PTSD, hippocampal neurogenesis

**This article has been corrected:** The authors found that the data used for statistical analysis of BrdU-positive cells in the DG region presented as a bar graph in **Figure 5C** is an accidental duplication of the bar graph in **Figure 5G**, which presents data used for statistical analysis of BrdU-positive cells in the DG region in a different experiment. The authors replaced the bar graph in **Figure 5C** with the correct data from the original corresponding experiment. This correction does not affect the article's conclusions.

Corrected **Figures 5** is presented below.

**Figure 5:**
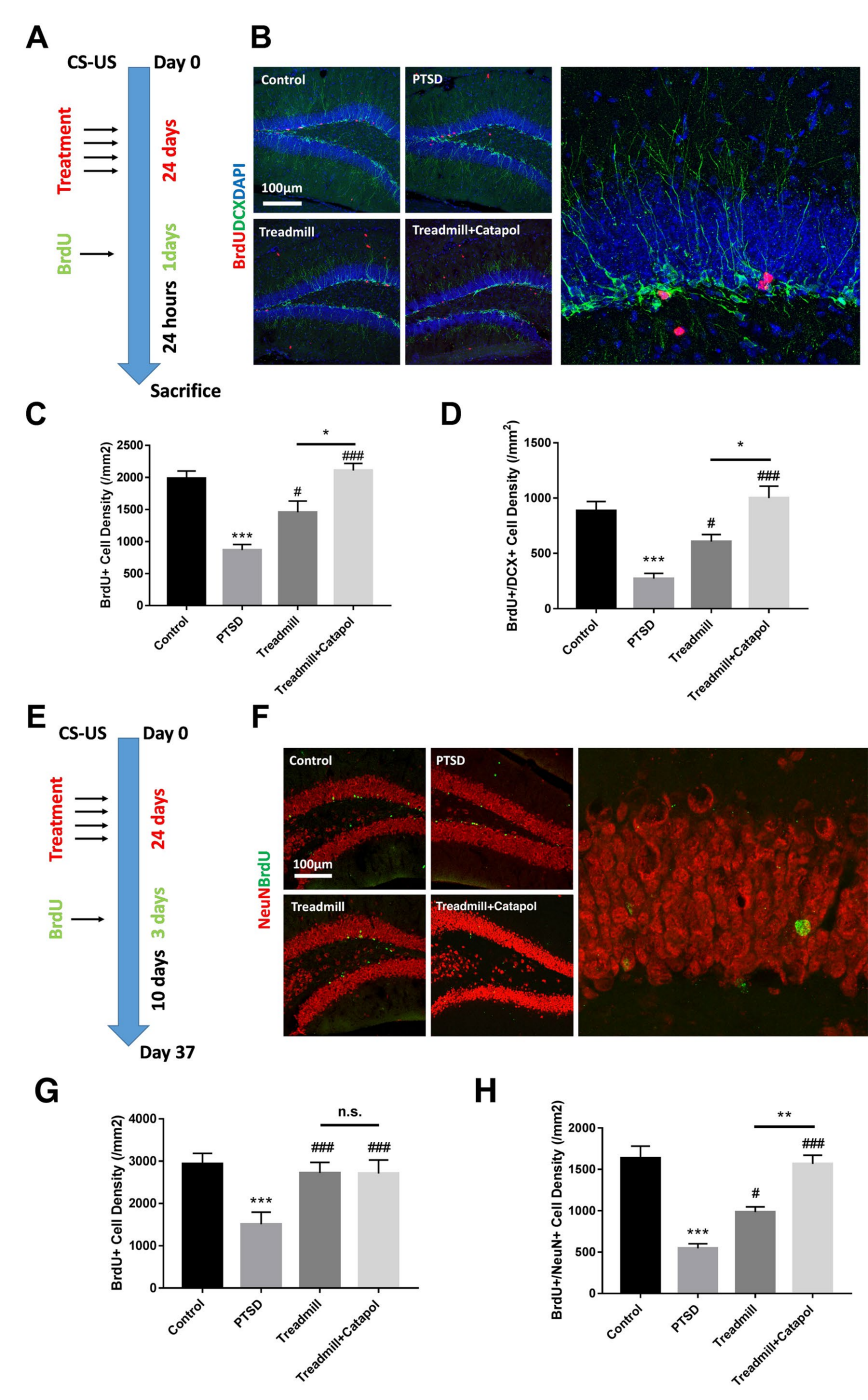
**Catalpol promoted neural differentiation without changing the survival of the immature neurons.** (**A**, **E**) experimental procedure of different BrdU injection protocol. (**B**) DCX staining (green) coupled with BrdU (red) in DG to assess the NSCs neural differentiation. (**C**, **D**) Statistical analysis of the BrdU positive cell and BrdU/DCX dual positive cells in DG region. (**F**) NeuN staining (red) coupled with BrdU (green) in DG to assess the neural maturation in DG. (**G**, **H**) Statistical analysis of the BrdU positive cell and BrdU/NeuN dual positive cells in DG region. One-way ANOVA, ***p < 0.001 vs. control; ^#^*p* < 0.05, ^###^*p* < 0.001.

